# Anal intraepithelial neoplasia screening during colonoscopy: a technical proposal

**DOI:** 10.1055/a-2387-4051

**Published:** 2024-09-04

**Authors:** Willian Ferreira Igi, Daiane Marrai Costa Nascimento, Magda Priscila Cardoso Afonso, Fernando Lander Mota, Lucas Santana Nova da Costa, Eloisa Barbosa Brum, Fernanda Freitas Franca Rocha

**Affiliations:** 1 Departamento de Gastroenterologia, CAEDRO – Centro Avançado de Endoscopia Digestiva de Rondônia, Porto Velho, Brazil; 2 67766 Endoscopy Department, Hospital de Amor, Barretos, Brazil; 3 42522 Endoscopy Unit, Hospital Sírio-Libanês, São Paulo, Brazil; 4 Endoscopy Unit, Hospital Sírio-Libanês, Brasília, Brazil; 5 Serviço de Coloproctologia, Hospital Barão de Lucena, Recife, Brazil; 6 Gastroenterology Department, 6. Hospital das Clínicas da Faculdade de Medicina de Ribeirão Preto, Ribeirão Preto, Brazil

Current recommendations for anal cancer screening are limited to high risk populations, utilizing anal cytology, high risk human papillomavirus (HPV) testing, and high-risk HPV-cytology co-testing. Depending on the results, patients are then referred for further evaluation with high-resolution anoscopy (HRA), a resource that is scarce in most locations 1 . This procedure can identify anal intraepithelial neoplasias (AINs), precursor lesions of anal squamous cell carcinoma, allowing for early treatment 2 .

Magnifying or image-enhanced endoscopies provide superior magnification compared to HRA 2 , and a classification system for AIN has even been proposed 3 . However, blind anorectal intubation remains a common practice during colonoscopy 4 , missing the opportunity to diagnose AIN.

In this video, we demonstrate the inspection of the anal canal using narrow-band imaging (NBI) and near focus with an Evis EXERA III CV-190 processor, Evis EXERA III CLV-190 light source, and CF-HQ-190 colonoscope (Olympus Medical Systems, Tokyo, Japan). The goal is to detect AIN, with a special focus on identifying abnormal intrapapillary capillary loops ( Fig. 1 , Fig. 2 ). Given the short length of the anal canal, its inspection can be quickly performed before the colonoscopy ( Video 1 ). The proximity of the canal walls makes the use of near focus or magnification highly advantageous, allowing for the detection of lesions that are often underdiagnosed.

**Fig. 1:**
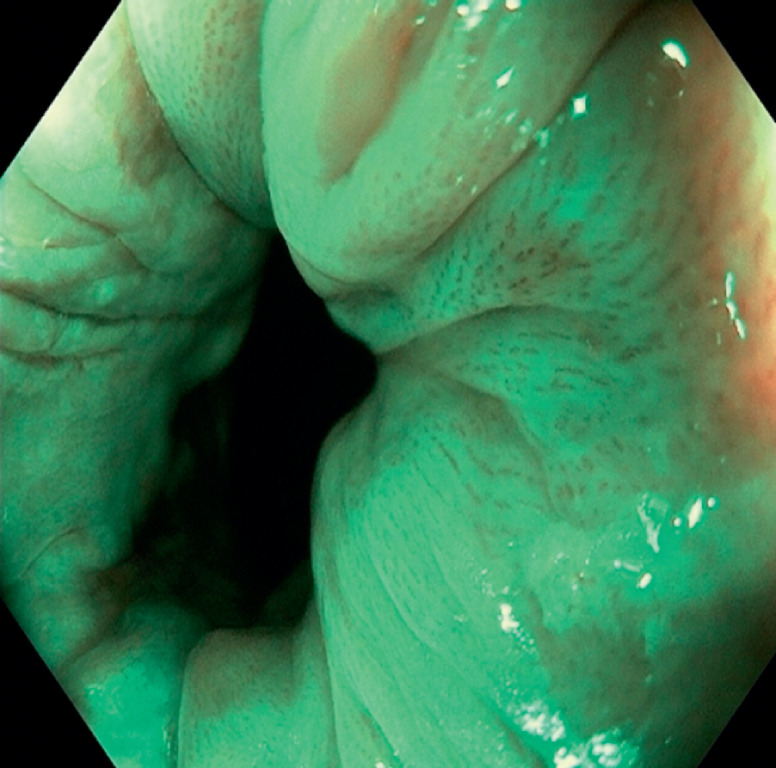
Low grade dysplastic lesion of the anal canal detected via colonoscopy with narrow-band imaging (NBI) and near focus.

**Fig. 2:**
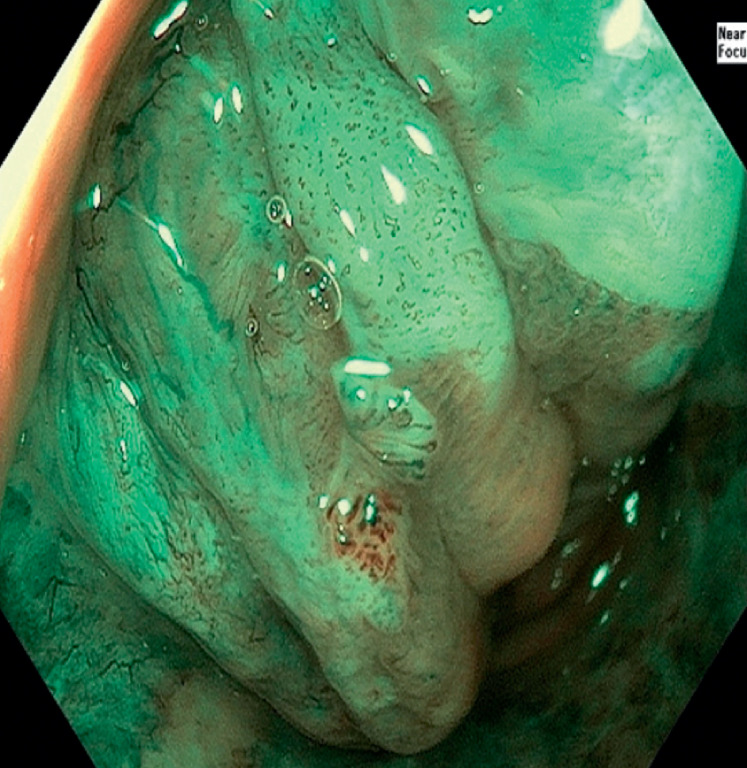
High grade dysplastic lesion of the anal canal with dilated, tortuous, and meandering intrapapillary capillary loops assessed using a disposable anoscope and NBI and near focus.

Anal evaluation during colonoscopy with the aim of identifying anal intraepithelial neoplasia.Video 1

Although there are limitations in the evaluation of the anal canal, the squamocolumnar junction can still be reasonably observed through insufflation or retroflexion 2 . After identifying the suspicious lesion, we chose to complement the examination with a disposable anoscope to achieve better visualization and stabilization for performing biopsies.

In this way, we consider that anal canal evaluation should be performed during all colonoscopies using NBI and near focus/magnification, aiming to identify AIN.

Endoscopy_UCTN_Code_TTT_1AQ_2AI

## Notes

Endoscopy E-Videos https://eref.thieme.de/e-videos
*E-Videos* is an open access online section of the journal *Endoscopy* , reporting on interesting cases and new techniques in gastroenterological endoscopy. All papers include a high-quality video and are published with a Creative Commons CC-BY license. Endoscopy E-Videos qualify for HINARI discounts and waivers and eligibility is automatically checked during the submission process. We grant 100% waivers to articles whose corresponding authors are based in Group A countries and 50% waivers to those who are based in Group B countries as classified by Research4Life (see: https://www.research4life.org/access/eligibility/ ). This section has its own submission website at https://mc.manuscriptcentral.com/e-videos .

## References

[1] Stier EA Clarke MA Deshmukh AA International Anal Neoplasia Societyʼs consensus guidelines for anal cancer screening Int J Cancer 2024 154 1694 1702 10.1002/ijc.34850 38297406

[2] Sakamoto T Akiyama S Narasaka T Anal intraepithelial neoplasia: Precursor of anal squamous cell carcinoma J Anus Rectum Colon 2022 6 92 99 10.23922/jarc.2021-077 35572484 PMC9045852

[3] Pecere S Hassan C La Milia D Accuracy of narrow-band imaging in predicting the histology of anal intraepithelial lesions Eur J Gastroenterol Hepatol 2023 35 31 35 10.1097/MEG.0000000000002457 36468566

[4] Martínez-Alcalá A Manovski K Mönkemüller K Direct anorectal intubation during colonoscopy: a logical new paradigm Endoscopy 2023 55 E488 E489 10.1055/a-2025-0284 36858351 PMC9977565

